# Differential sensitivity of bees to urbanization-driven changes in body temperature and water content

**DOI:** 10.1038/s41598-018-38338-0

**Published:** 2019-02-07

**Authors:** Justin D. Burdine, Kevin E. McCluney

**Affiliations:** Department of Biological Sciences, Bowling Green State University, Bowling Green, OH USA

## Abstract

Predicting how species will respond to climate change and land use modification is essential for conserving organisms and maintaining ecosystem services. Thermal tolerances have been shown to have strong predictive power, but the potential importance of desiccation tolerances have been less explored in some species. Here, we report measurements of thermal and desiccation tolerances and safety margins across a gradient of urbanization, for three bee species: silky striped sweat bees (*Agapostemon sericeus*), western honeybees (*Apis mellifera*), and common eastern bumblebees (*Bombus impatiens*). We found significant differences in thermal tolerances, measured as critical thermal maximum (CT_max_), amongst species. Bumblebees were the least sensitive to warming, with a higher CT_max_ (53.1 °C) than sweat bees (50.3 °C) and honeybees (49.1 °C). We also found significant differences in desiccation tolerances, measured as critical water content (CWC), between all species. Sweat bees were the least sensitive to desiccation, with the lowest CWC (51.7%), followed by bumblebees (63.7%) and honeybees (74.2%). Moreover, bumblebees and sweat bees were closer to their CT_max_ in more urbanized locations, while honeybees were closer to their CWC. These results suggest that bees have differential sensitivities to environmental change and managing for diverse bee communities in the face of global change may require mitigating both changes in temperature and water.

## Introduction

Climate change and land use modification can have negative consequences for many species, leading to local population declines^1–3^ and extinctions^4^. When certain taxa decline, the services they provide (e.g. pollination) can be disturbed or degraded^5,6^. While examples of population declines with climate change and land-use modification are accumulating^1–3,7^, proximate mechanisms mediating these declines are often unclear because very species-specific patterns occur. Understanding these mechanisms is vital to identifying actions that mitigate the potential losses of ecosystem services. Multiple studies have identified the importance of physiological tolerances in predicting species responses to global change^3,8–11^, as these tolerances demarcate the environmental conditions necessary for survival. However, the majority of studies investigating physiological tolerances focus on thermal tolerances^12–14^, while desiccation tolerances may be just as important.

Thermal tolerances are an important tool for investigating species responses to changes in temperature^15–17^. Critical thermal maximum (CT_max_) and minimum (CT_min_), and thermal safety margins are the most common metrics of physiological vulnerability to climate change for a variety of organisms^13,18,19^. CT_max_ is an organism’s upper sub-lethal temperature and CT_min_ the lower sub-lethal temperature^20^, and these are the temperatures at which an organism loses muscular control and suffers an ecological death. The difference between CT_max_ and CT_min_ is defined as the thermal range^21^. Thermal safety margin is defined as the differences between CT_max_ and either optimal body temperature, field body temperature, or air temperature, and offers a metric for understanding vulnerabilities to warming^8^. In general, thermal tolerance has been found to vary with natural temperature gradients. Insects from high and low latitudes tend to have similar CT_max_ values, while CT_min_ declines with latitude^18,22^ and thermal safety margin increases with latitude^18^. CT_max_ has been shown to decreases with altitude^13,23^. There is also evidence that insect thermal tolerance varies across smaller climatic gradients^24^. Body size (surface-volume ratios) may also influence thermal tolerance, because smaller animals dissipate heat better but may be more prone to desiccation^25^.

Desiccation tolerances may also be an important mediator of climate effects on species^26^, but few studies have explored desiccation in this context^27–30^, and little information exists about desiccation tolerance of animals in general^31–35^. Hadley^34^ reported that the majority of arthropods maintain a water content between 65 and 75%, while some arthropods may survive with a water content as low as 40%^31^. We know little about how desiccation tolerance varies with environmental gradients, but Hoffman *et al*.^27^ suggest that fruit fly desiccation tolerance declines with increasing precipitation in Australia. Weldon *et al*.^36^ provide evidence that desiccation tolerance varies geographically among populations of Mediterranean fruit flies. Moreover, McCluney *et al*. (2017) found that arthropod hydration decreased with urbanization and consequent warming in moist/mild cities (Raleigh, NC), but sometimes increased with urbanization in warmer cities (Phoenix, AZ; Orlando, FL). The observed variations in desiccation tolerance may be due to local adaptations, or plasticity^37^. These studies suggest that desiccation tolerance might be an important predictor of the effects of land use and climate change on animals. Desiccation may be particularly likely for smaller animals, like insects, due to their greater surface area to volume ratios and higher water loss relative to metabolic rate^38^.

Multiple metrics of desiccation tolerance have been employed by others. Many studies have used the time until 50% of the animals die (LT50)^27,39^ for both desiccation and thermal tolerance measurements. However, another measure, body water content at death (critical water content, CWC), provides values comparable across studies. CWC is an experimental measurement of desiccation tolerance, or the lethal water content, calculated gravimetrically as the difference between wet and dry mass, divided by wet mass^40^. Unlike thermal safety margins, none have calculated a *hygric safety margin* (hygric = relating to moisture), which we have used in this study and define here as the difference between CWC and field body water content. This metric could be highly informative in predicting potential responses to climate change, complementing thermal analogs.

Urban environments are unique systems for examining how land use modification and climate change influence thermal and desiccation tolerances^41^. Summer urban heat islands (UHIs) in the USA typically generate a mosaic of hotter and cooler locations that differ, on average, by 1–4 °C from each other and form cooler, rural temperatures^42^. The intensity of UHIs is often influenced by the biome in which the city is located. Urban areas within regions dominated by temperate and mixed forest can experience temperature increases of 8 °C, while urban areas within desert regions experience less pronounced changes or temperature decreases in urban centers^43^. UHIs provides a gradient of temperatures^44,45^ that can replicate projected climate change^41^. Urban areas can also experience altered soil moisture^46^, and soil moisture is found to vary among habitat types within and among cities^47^. These changes in temperature and moisture availability can impact the field body water content of arthropods^48^. A recent study by Hamblin *et al*. (2017) found CT_max_ to predict population change for 15 bee species across an urbanization gradient. We know relatively little about bee thermal tolerances^13,15,49,50^, and even less about the desiccation tolerances of bees^49^, even though these factors may provide strong predictive power in explaining population declines or changes in distributions. Elevated temperatures due to UHI effects are likely to increase desiccation threats. Others have found that changes in insect water balance can have consequences on growth, reproduction, and survival^51^.

Here we examine how a gradient of urbanization (impervious surface, e.g. areas of pavement), in a medium-sized city, alters both the CT_max_ and CWC of three bee species: the silky striped sweat bee (*Agapostemon sericeus*), the western honeybee (*Apis mellifera*), and the common eastern bumblebee (*Bombus impatiens*). We combine measurements of thermal and water content limits with measurements of field body temperature and field water content, to quantify thermal and hygric safety margins. We test the relative importance of two competing hypotheses about limits and safety margins. First, thermal and water content limits may change rapidly within taxa due to changes in temperature and moisture conditions, with sensitivity decreasing with urbanization-induced warming and drying. In this case, thermal and hygric safety margins would not vary with urbanization because limits change in concert with field body temperature and water content (“*shifting limits hypothesis*,” i.e. limits are plastic). Second, thermal and water content limits may change slowly within taxa, being insensitive to urbanization-induced warming and drying. In this case, limits would not change with urbanization, even as average field body temperature or water content did change, and we would expect safety margins to be related to urbanization-induced warming or drying (“*stable limits hypothesis*,” i.e. limits are non-plastic).

## Materials and Methods

### Study Area

**Figure 1:**
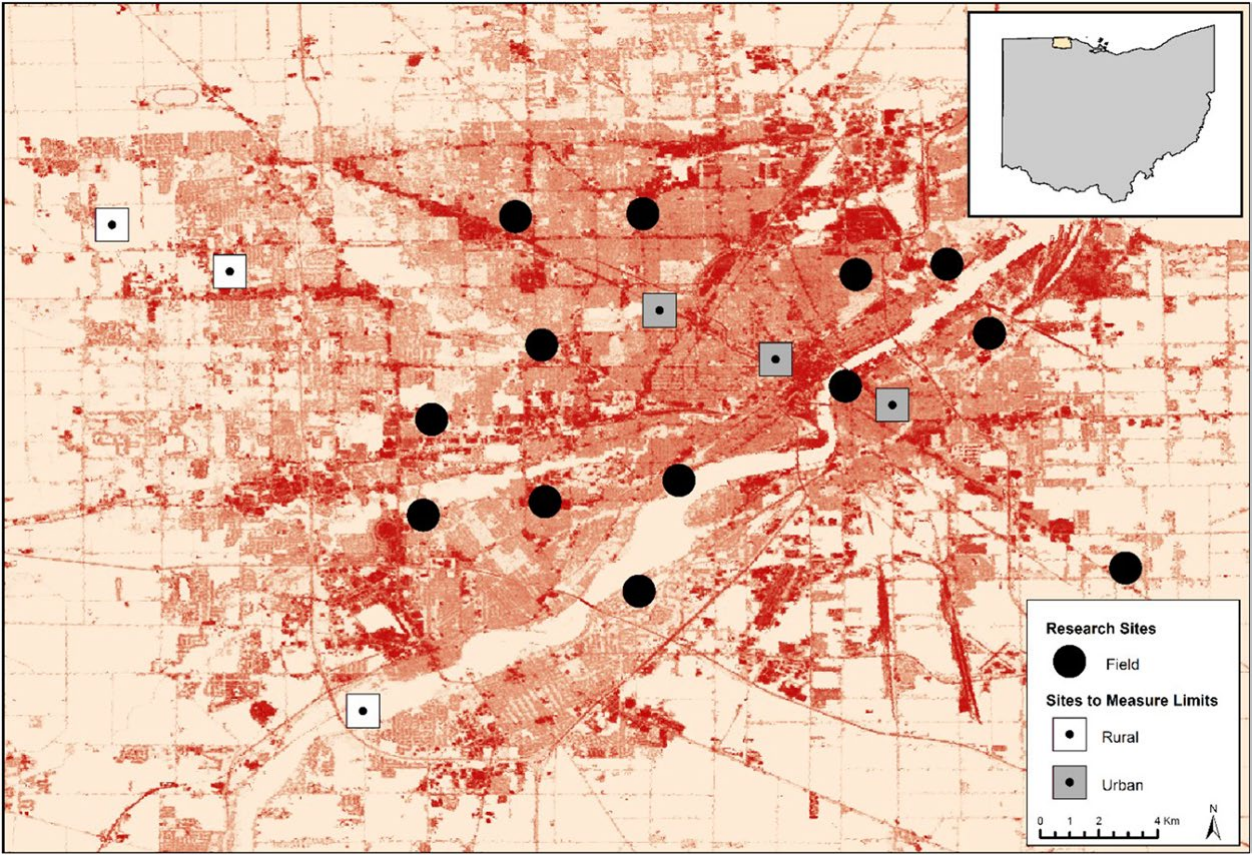
Map displaying site locations in Toledo, Ohio, with reference to the state of Ohio. Base layer coloration indicates percent impervious surface. Darker shades of red represent high impervious surface, and light shades represent low impervious surface. CT_max_ and CWC were determined for square sites (3 urban, 3 rural). Field measurements body temperature and water content were measured at all 19 sites. This map was created using the 2011 National Landcover Dataset percent developed imperviousness layer^54,81^ in ArcGIS 10.3.1.

Field body temperature and water content were measured in bees collected from 19 sites, and CT_max_ and CWC were measured on bees collected from a subset of sites, across the metropolitan region of Toledo, Ohio, USA (Fig. 1). Toledo has been shown to display urban heat island effects with 2 °C warmer mean annual temperatures^52^. Toledo has a 622.6 square-kilometer metropolitan area with a population of 507,643 residents and a large network of greenspaces (125 city parks and 150 urban gardens)^53^ that were used as sampling locations^52^ Sites were selected by overlaying a grid over a map of Toledo in ArcGIS and numbering each grid cell (2 × 2 km). A random number generator was used to identify grid cells for sampling, and we chose a park or garden within each selected cell. We quantified imperviousness (land surfaces that prevent water infiltration) for each site using the percent developed imperviousness layer in the 2011 National Land Cover Dataset^54^ in ArcMap 10.3. We calculated local imperviousness (300 m radius) and landscape imperviousness (2000 m radius), as scale can be important in appropriately measuring the urban landscape for bees^55^.

### Study Organisms

We focused on the silky striped sweat bee (*Agapostemon sericeus*), western honeybee (*Apis mellifera*), and common eastern bumblebee (*Bombus impatiens*) due to the relative commonness of these species in parks and gardens across Toledo^56^. In general, sweat bees begin their activity in early to mid-Spring when temperatures reach 14 °C^57^, while bumblebees and honey bees are known to forage at much lower temperatures^58^. Most research on thermal tolerance in bees is focused on bumblebees and honeybees, and we included sweat bees because little is known of their physiological tolerances, they are abundant and widely distributed, and they are smaller than the other species. These three species differ in size, foraging preference, sociality, and nest specificity^59^, increasing the likelihood of detecting differential responses among species.

### Field Body Temperature and Water Content

We hand collected sweat bees, honeybees, and bumblebees from a series of 19 sites from June-August 2016 using 120 ml specimen containers (QuickMedical Model#4256). Bees were sampled in the mornings on days with clear skies and temperatures above 22 °C. Upon capture, bees were anesthetized with CO_2_ to estimate body temperature using a thermocouple (Atkins 351/352) held between the thorax and abdomen for approximately 30 s. Surface temperatures of bees and other insects, at rest, have been found to be similar to internal body temperatures (within 1 °C)^60,61^. We then transported bees back to the lab in airtight vials (Pelco® Mini Vials) to prevent water loss, and placed the vials in a freezer for 24 hours prior to water content determination. Bee water content was calculated gravimetrically using an analytical balance (Mettler Toledo XPE56) with precision to one microgram. We calculated the water content of each bee as the difference in wet and dry mass divided by wet mass. To calculate dry mass, bees were placed into a drying oven (Thermo Fisher Scientific #151030520) set at 55 °C for at least 48 hours, and then reweighed^48^. We obtained estimates of water content from all 19 sites, but our temperature measurements are from only 13 sites. We were unable to take body temperature measurements at all 19 sites due to equipment availability.

### Thermal and Water Content Limits Sampling

To measure thermal and water content limits, we focused on 6 sites (3 urban, 3 rural) in the metropolitan region of Toledo (Fig. 1), that were selected based on the percent impervious surface within a 300 m radius of the site center (see Fig. 1). Urban sites had an average local impervious surface (300 m) of 57.79% ± 9.6, while rural sites had an average local impervious surface of 23.24% ± 4.5. For each experiment, we collected 15 bees from each site of the same species identified above (five sweat bees, five honeybees, five bumblebees). At each site, we walked a linear transect starting at the site’s center and collected the first five bees of each species we encountered using 120 ml specimen containers. Upon collection, bees were placed in a cooler box without ice (25 °C) to standardize temperatures prior to each experiment, and immediately taken to Bowling Green State University. Bees were given a 1 M sucrose solution upon capture to ensure bee survival during transportation prior to thermal ramping and desiccation trials, which was especially important to ensure survival of honeybees^50^. All experimental trials began within two hours of collection. The collection period for bees in our thermal and hygric experiments occurred during September 6–22, 2016.

### Thermal Tolerance Experiment

We measured critical thermal maximum (CT_max_) for each sampled bee with a temperature ramping experiment using an environmental chamber (Memmert HPP 750). The temperature ramp began at 25 °C and increased at a rate of 0.5 °C min^−1^, following standard methods^20^. Humidity was held constant at 30%. We used iButton® temperature and humidity data loggers (DS1923) to verify the conditions of the environmental chamber. Bees were placed individually into a 120 ml specimen cup (QuickMedical Model#4256) with a mesh cover that allowed air temperatures within specimen cups to increase with the temperature ramp. Additionally, the mesh top allowed us to easily test the righting response using a puff of air (Giottos AA1900). The loss of righting response indicates an endpoint when muscle functions begin to fail, and is commonly used to estimate CT_max_^12,13^. Bees unable to flip themselves upright within 15 seconds after receiving a puff of air were considered to have lost the righting response. The temperature at which the righting response was lost was recorded as the CT_max_, and bees were removed from the environmental chamber after this point. The temperature ramp ended when all 90 bees had reached their CT_max_ (~2 hrs). All samples were immediately weighed and stored in air-tight vials after the temperature ramp ended. Bees were not given food or water during the temperature ramping period.

### Desiccation Tolerance Experiment

We measured critical water content (CWC) by placing a different set of 90 bees in a desiccation cabinet at 0% humidity and a constant temperature of 25 °C (well below CT_max_). Humidity was maintained at 0% using Drierite (#60011T), and was verified using an iButton® temperature and humidity logger (DS1923). Bees were placed individually into the same 120 ml specimen cups used in the CT_max_ experiment and checked on an incremental timescale as follows: (1) every 15 minutes, (2) every 30 minutes, (3) every 1 hour, (4) every 3 hours, (5) every 6 hours, and (6) every 12 hours. We performed three checks of each time period. Each check lasted approximately five minutes, and we tested the bees’ righting response and removed unresponsive bees. Immediately after removal from the desiccation cabinet, bees were placed into pre-weighed Pelco® Minivials for water content determination.

### Thermal and Hygric Safety Margins

We calculated thermal and hygric safety margins as measures of the proximity of bees to limits^8^. Thermal safety margins were calculated as the difference in the measured field body temperature and the 90^th^ percentile CT_max_. Hygric safety margins were calculated similarly as the difference in the measured field water content and the 10^th^ percentile CWC. Using 90^th^ and 10^th^ percentiles, respectively, prevented us from using abnormally tolerant individuals to estimate safety margins, while simultaneously preventing negative safety margins (see Mccluney *et al*. 2017) that might have arisen had we used mean CT_max_ or CWC values.

### Statistical Methods

All statistical tests were conducted in the R statistical environment (version 3.1.3). We used the *nlme* package^62^ to fit linear mixed effects models (*lme*), comparing CT_max_ and CWC among bee species (*A*. *sericeus*, *A*. *mellifera*, *B*. *impatiens*) and site class (rural, urban), and the interaction effect between species and site class. We included site ID as a random effect to account for inclusion of multiple bees per site. Post-hoc Tukey multiple comparison of means (*glht*) tests, in the *multcomp* package^63^, were used following significant main effects.

Linear mixed effects models were also used to examine relationships between thermal and hygric safety margins and our urbanization metrics (local imperviousness, landscape imperviousness), with site as a random effect to account for inclusion of multiple bees per site. We also used linear mixed effects models to test for relationships between safety margins and bee mass, including site and bee species as random effects. We tested for the significance of fixed effects via likelihood ratio tests with removal of the fixed effect from the model^64^. Significance was indicated and discussed at α = 0.05 for a test of the core hypotheses, and at α = 0.1 as a threshold for patterns that could be further explored in the future. Assumptions of normality were checked via examination of plots of residuals, and measures of hygric safety margins were logit transformed.

## Results

### Critical Thermal Maximum (CT_max_)

**Figure 2:**
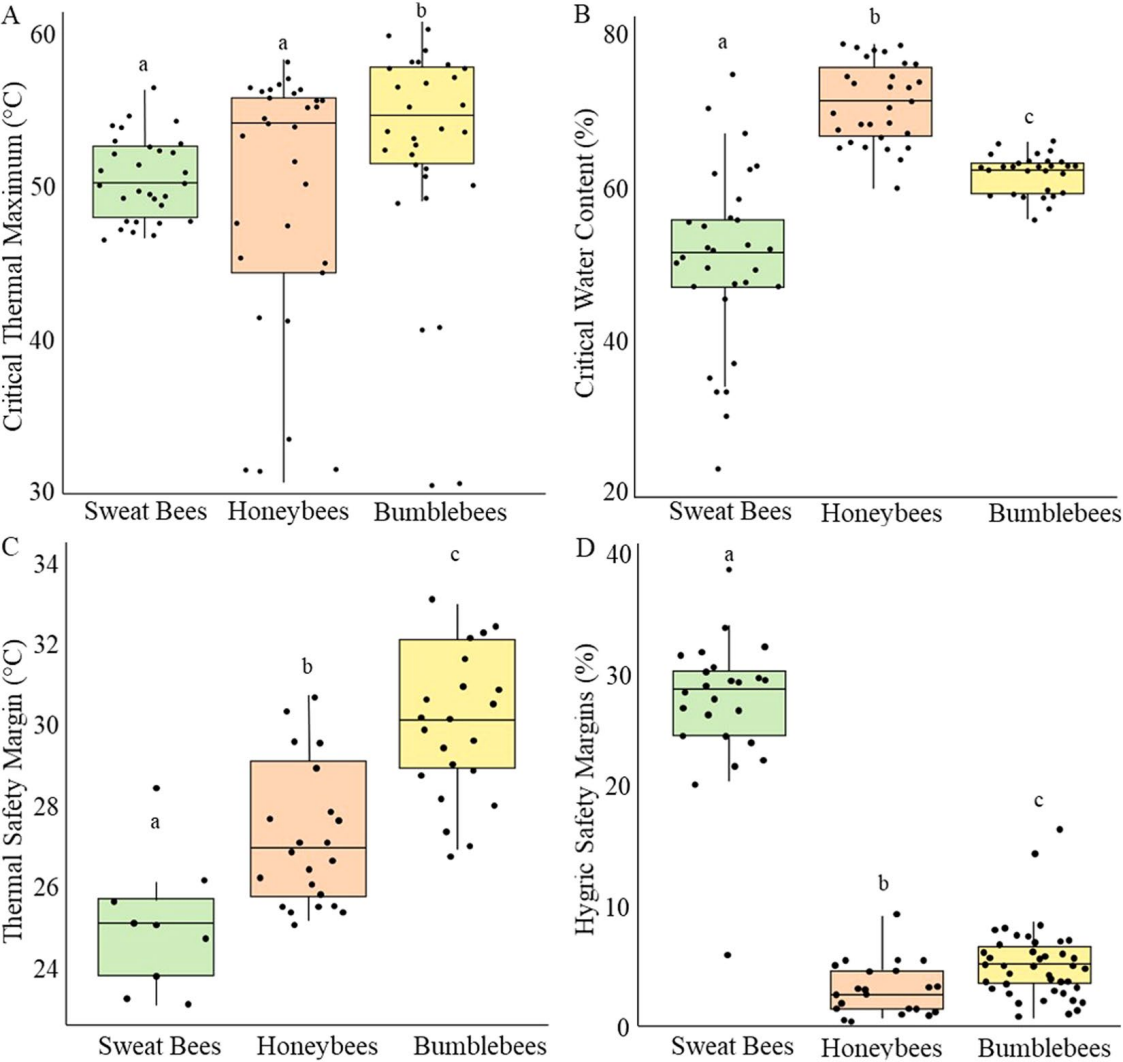
Differences across bee species for thermal and hygric limits. (**A**) CT_max_ was significantly different between species (χ^2^ = 6.56; p = 0.038), and bumblebees had a higher CT_max_ than honeybees and sweat bees; (**B**) CWC was significantly different between all species (χ^2^ = 77.81; p < 0.001) and sweat bees had the lowest CWC; (**C**) thermal safety margins were significantly different across all species (χ^2^ = 55.62; p < 0.001), and honeybees had the largest thermal safety margin; and (**D**) hygric safety margin was significantly different across all species (χ^2^ = 97.433; p < 0.001), and sweat bees had the largest hygric safety.

We measured CT_max_ for 88 bees (30 sweat bees, 30 honeybees, 28 bumblebees). Our measurements varied significantly between species (df = 2, χ^2^ = 6.56, p = 0.038; Fig. 2A). A post-hoc test revealed that bumblebees had significantly higher CT_max_ than honeybees and sweat bees, but there was no difference between honeybees and sweat bees. Although we did not detect a significant difference in CT_max_ between urban and rural sites at α = 0.05 (df = 1, χ^2^ = 3.84, p = 0.05; S1), a strong trend was apparent in mean CT_max_ between urban (52.0 °C ± 0.76) and rural sites (49.6 °C ± 1.1). We found no difference in the slope of the relationship between urbanization and CT_max_ between species (Urbanization x Species: df = 2, χ^2^ = 0.745, p = 0.69). We also found no significant relationship between CT_max_ and bee mass (df = 1, χ^2^ = 2.357, p = 0.125, R^2^ = 0.02). Bee mass did not differ between urban and rural sites (df = 1, χ^2^ = 0.488, p = 0.485), but we did find significant differences in mass between species as expected (df = 2, χ^2^ = 139.8, p < 0.001). Bumblebees had the largest mass (44.97 mg ± 2.81), followed by honeybees (31.39 mg ± 0.46), and sweat bees (4.00 mg ± 0.44).

### Critical Water Content (CWC)

We measured CWC for a different set of 89 bees (30 sweat bees, 30 honeybees, 29 bumblebees), and our measurements varied significantly between species (df = 2, χ^2^ = 77.81, p < 0.001; Fig. 2B). A post-hoc multiple comparisons test showed significant differences in CWC between all bee species. Sweat bees had the lowest CWC (highest tolerance), and honeybees the highest CWC (lowest tolerance). However, we did not detect a significant difference in CWC between bees from urban and rural sites (df = 1, χ^2^ = 3.26, p = 0.071), and we found no interaction between species and urbanization (df = 2, χ^2^ = 2.44, p = 0.29). We also found no relationship between CWC and bee mass (df = 1, χ^2^ = 1.94, p = 0.16; R^2^ < 0.001).

We also found significant differences between species in the length of time they survived in the desiccation cabinet (df = 2, χ^2^ = 9.87, p = 0.007). A post-hoc multiple comparisons test found sweat bees (39.5 hours ± 8.03) to have longer survival times than honeybees (12.3 hours ± 6.85) (p = 0.005), and statistically similar survival times to bumblebees (19.5 hours ± 2.65) (p = 0.057). We found no differences in survival times between honeybees and bumblebees (p = 0.70). We also found no differences in survival times between bees collected from urban and rural sites (df = 1, χ^2^ = 0.71, p = 0.40).

### Thermal Safety Margin

**Figure 3:**
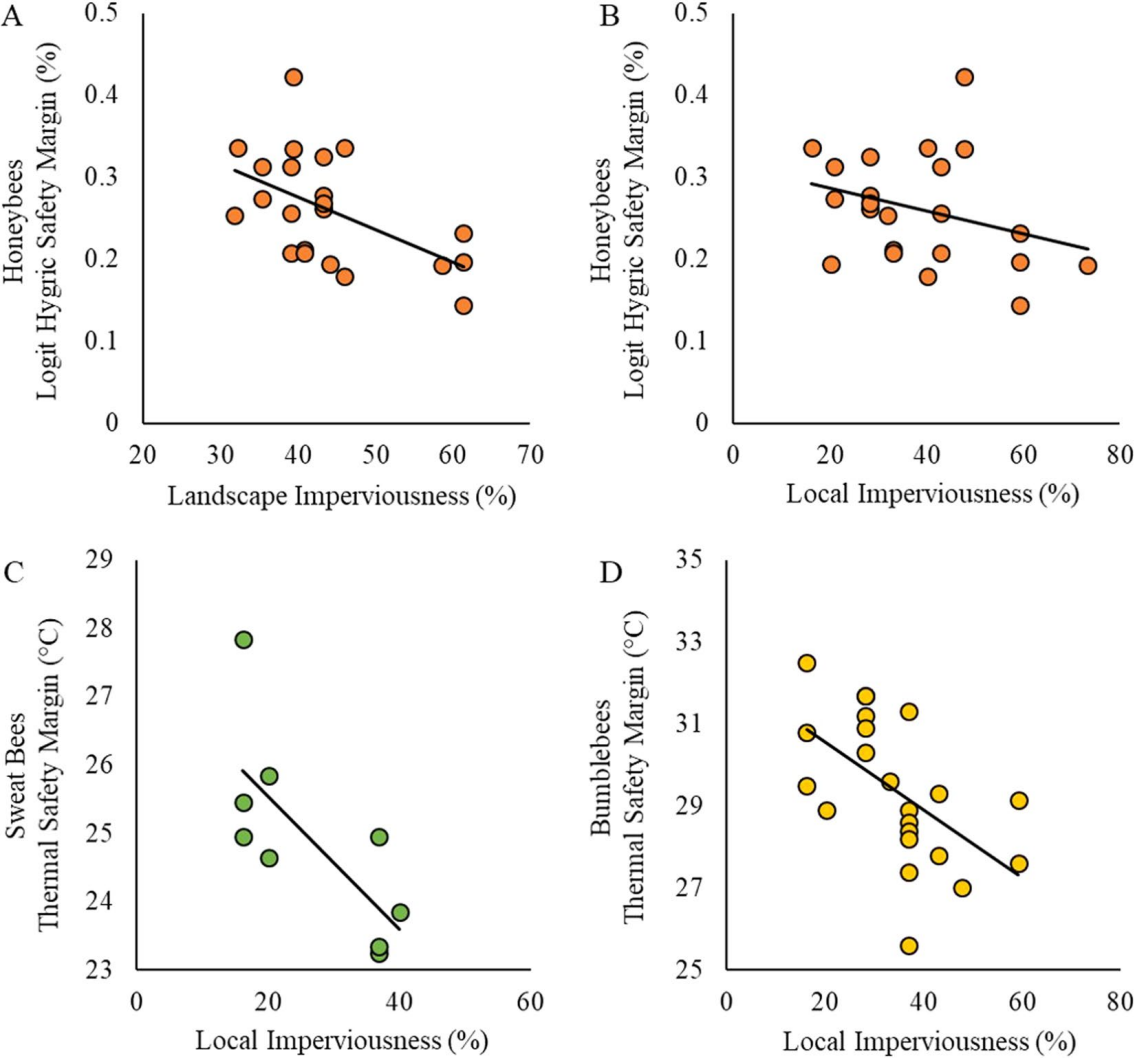
**S**ignificant relationships between safety margins (thermal and hygric) and imperviousness (local and landscape) for each bee species. (**A**) Honeybee hygric safety margin is significantly associated with landscape imperviousness at α = 0.05 (χ^2^ = 8.847, p = 0.003, R^2^ = 0.19); (**B**) Honeybee hygric safety margin is also associated with local imperviousness at the α = 0.1 level (χ^2^ = 2.81, p = 0.094, R^2^ = 0.04); (**C**) Sweat bee thermal safety margin is significantly associated with local imperviousness (χ^2^ = 6.28, p = 0.012, R^2^ = 0.49); and **(D**) Bumblebee thermal safety margin is significantly associated with local imperviousness (χ^2^ = 4.905, p = 0.027).

We calculated thermal safety margins for 52 bees (22 bumblebees, 21 honeybees, 9 sweat bees) taken from 13 sites that varied in the amount of impervious surface present around our collection sites (our metric of urbanization) at the local (300 m radius) and landscape (2 km radius) scale. Thermal safety margins declined with increasing impervious surface, but bee species differed in the strength of this response (Table 1). Overall, bumblebees (29.38 °C ± 0.38) had the largest thermal safety margin followed by honeybees (26.95 °C ± 0.37) and sweat bees (24.91 °C ± 0.47). We found thermal safety margin declined with local (300 m) imperviousness for both bumblebees (Fig. 3D) and sweat bees (Fig. 3C), but not for landscape imperviousness. For honeybees, thermal safety margin was not associated with imperviousness at either scale. Overall, we found no relationship between thermal safety margin and bee mass (df = 1, χ^2^ = 1.93, p = 0.165, R^2^ = 0.02).

**Table 1:** Results of linear mixed effects models comparing thermal and hygric safety margins to local (300 m) and landscape (2 km) imperviousness.

Model Component Removed	df	ΔAIC	LRT(χ^2^)	P-Value
**Hygric Safety Margin ~ Local**				
Species * Local	2	204.51	3.361	0.186
**Species**	**2**	**296**.**09**	**95**.**584**	<**0**.**001**
Local	1	205.47	2.962	0.085
**Hygric Safety Margin ~ Landscape**				
Species * Landscape	**2**	**200**.**85**	**9**.**424**	**0**.**01**
**Species**	**2**	**294**.**28**	**97**.**433**	**<0**.**001**
**Landscape**	**1**	**205**.**47**	**6**.**623**	**0**.**01**
**Thermal Safety Margin ~ Local**				
Species * Local	2	183.47	0.457	0.796
**Species**	**2**	**235**.**09**	**55**.**623**	**<0**.**001**
**Local**	**1**	**186**.**42**	**3**.**951**	**0**.**047**
**Thermal Safety Margin ~ Landscape**				
Species * Landscape	2	186.67	0.570	0.752
**Species**	**2**	**235**.**51**	**52**.**842**	**<0**.**001**
Landscape	1	185.42	0.749	0.387

Each model begins with interaction terms, followed by the effect of each component removed individually. Significant results with a p-value < 0.05 are in bold.

### Hygric Safety Margin

We calculated hygric safety margins for 88 bees (42 bumblebee, 24 sweat bees, 22 honeybees) taken from 19 sites that varied in the amount of impervious surface present around our collection sites (our metric of urbanization). Hygric safety margin varied interactively with imperviousness and bee species at the landscape level (df = 2, χ^2^ = 9.014, p = 0.01; Table 1). Sweat bees had the largest hygric safety margin (26.15% ± 1.22), followed by bumblebees (5.25% ± 0.46) and honeybees (2.50% ± 0.45). For honeybees, hygric safety margin declined with landscape imperviousness at α = 0.05 (df = 1, χ^2^ = 8.847, p = 0.003; Fig. 3A), with a similar trend for local imperviousness at α = 0.1 (df = 1, χ^2^ = 2.81, p = 0.094; Fig. 3B). For sweat bees and bumblebees, we found no associations between imperviousness and hygric safety margin. Overall, we found no significant relationship between hygric safety margin and bee mass (df = 1, χ^2^ = 0.06, p = 0.807; R^2^ < 0.001). Field water content did not differ between urban and rural sites (df = 1, χ^2^ = −311.34, p = 0.91), but we did find significant differences in field water content between species (df = 2, χ^2^ = −253.04, p < 0.001). Sweat bees had the lowest field water content (59.3% ± 1.2), followed by bumblebees (65.6% ± 0.4), and honeybees (70.1% ± 0.46).

## Discussion

Overall, we found that bee species differ in their thermal limits and water content limits, and their proximity to those limits, with certain species (honeybees) being more susceptible to desiccation than to extreme temperatures. This points out the need to better investigate desiccation, along with temperature, as potential mechanisms underlying the biological effects of global change. We also found that all three species were closer to their physiological limits (either thermal or hygric) with increasing urbanization (local or landscape imperviousness), and that thermal and hygric limits did not vary significantly with urbanization, supporting the “stable limits hypothesis”.

The proximity of honeybees to their water content limits in highly urbanized areas likely represents a high risk for population declines during a drought or heat wave (due to increased water loss rates). However, given that bumblebees and sweat bees were not close to their thermal limits, and that physiological performance often increases as an animal approaches its thermal limits^18,65^, the increasing proximity to thermal limits for these two species may actually represent a benefit rather than an increased risk. Essentially, warming related to urbanization may benefit bumblebees and sweat bees in cool cities like Toledo, Ohio (<15 days per year above 32 °C), compared to many other US cities. However, we suggest that if increased proximity to thermal limits with urbanization also occurs in warmer cities, urbanization may represent substantial increased risk of population declines in those cities. This hypothesis needs further testing, but is supported by research from a warmer, southeastern US city, Raleigh, NC, USA, where critical thermal maximum (CT_max_) was a strong predictor of species presence with increased urbanization^15^.

### Thermal Tolerance

We found no significant differences in CT_max_ between urban and rural sites. Others have found CT_max_ to vary with natural temperature gradients, such as elevation^13^ and latitude^66^, and multiple studies have identified patterns between CT_max_ and urbanization^15,17,67,68^. For example, Diamond^17^ found differences in CT_max_ between urban and rural acorn ants, and attributed these differences to evolved plasticity to local environmental conditions. Angilletta^68^ found urban ants more heat tolerant than rural ants, but were unable to determine whether the differences were attributed to environmental or genetic effects. These studies seem to support the “shifting limits hypothesis”. We hypothesize that bee movement distances may help explain why we found stronger effects on safety margins than limits. Research suggests that bee body size predicts foraging distance^69^, and the species used in our study are relatively large bees. Greater mobility may prevent local adaptation or even local acclimation, as mobile species encounter both very urbanized and poorly urbanized landscapes, and have greater ability to evade lethal conditions. In addition, any small amount of local adaptation could be erased by genetic mixing. Results might be different for small bees (i.e. *Lasioglossum*) with short foraging ranges (sensu local adaptation was observed for acorn ants in Diamond^17^). In addition, results may be different with a larger sample size, or at sites with more extreme levels of impervious surface as others have found when using rural sites with 0% impervious surface^17^.

We note that bumblebees had higher CT_max_ than both honeybees and sweat bees, independent of urbanization. Others have found CT_max_ to increase with bee size^13^, although the exact mechanism is unclear. We found no significant relationship between bee mass and CT_max_, when controlling for species. Furthermore, sweat bees were the smallest bees measured and their CT_max_ was not significantly different from honeybees. Hamblin *et al*. found sociality to be an important factor in heat tolerance^15^, and our results offer some support for the importance of sociality in heat tolerance. Honeybees are eusocial and thermoregulation has been commonly observed in honeybee colonies^70,71^. While thermoregulation has also been observed for some bumblebee species^72,73^, we suggest that highly social honeybees may experience less selection pressure for increased CT_max_ than bumblebees, due to hive thermoregulation.

### Desiccation Tolerance

Although little research has been done on critical water content (CWC), the values we collected for bee water content are generally consistent with the reporting of other studies on arthropods^32–34,40^. While CWC values for sweat bees are consistent with the other arthropod taxa mentioned above, honeybees and bumblebees have much higher CWC. Other research has documented particularly high water loss rates in honeybees^49^, and here we find that they are relatively intolerant of desiccation. It is also interesting to note that honeybees and bumblebees have a tight range of tolerance, suggesting relatively little variation in water content limits in these populations.

Our research suggests key unanswered questions about CWC that future research should investigate. For instance, a better understanding of mechanistic drivers of variation in CWC is needed in general, including for bees. Following earlier work on other arthropods^34^, we suggest CWC may be associated with functional traits like cuticular hydrocarbon content, physiological stress response pathways, coloration, or other traits, with a variable degree of genetic influence. Moreover, our work suggests there may be a tradeoff between CWC and CT_max_ (bees were either more sensitive to heat or desiccation). But these ideas are in need of further, more explicit testing.

### Thermal and Hygric Safety Margins

We found evidence that all species of bees have decreased safety margins with increasing urbanization. At high impervious sites, bumblebees and sweat bees were both closer to their thermal limit, and honeybees were closer to their hygric limit. These results have several significant implications.

First, many studies have shown that urban areas are able to support diverse bee communities^74,75^, particularly bumblebees^55,76,77^. The observed positive effects are typically thought to be due to increased floral resources or nest site availability in urbanized regions^56,78,79^. However, our research suggests that associations between urbanization and bumblebee thermal physiology could be beneficial or harmful depending on the typical regional temperatures, altering thermal safety margins. Although bees function as facultative endotherms in flight, the body temperatures of flying bees increase with environmental warming^25^. Populations of both bumblebees and sweat bees may benefit from urbanization in cool regions, because higher ambient temperatures bring bees towards optimal operative temperatures. But these same species may be harmed by urbanization in warmer cities where bees are already near optimal operative temperatures. Likewise, in warm cities, these species may have reduced capacity to tolerate further increases in temperature associated with climate change or heat waves. Efforts to increase green spaces and shade within warm cities could help bees thermoregulate^14^ and reduce risk. Although we did not investigate CT_min_ (e.g. cold resistance) in this study, urban warming may lead to higher CT_min_ values and an overall decrease in thermal tolerance range, a as has been shown in ants^17^.

Second, we found that honeybees were closer to their hygric limits with increasing urbanization, causing them to be on the edge of their desiccation tolerance. Although bees drink a nectar diet and produce metabolic water, they can regularly become dehydrated in the field^25^. Water drinking behavior has been observed in honeybees^80^ and this is consistent with their low desiccation tolerance. Here we also show that honeybees are unable to maintain their water content with increasing urbanization, even in this relatively cool, mesic city. This suggests that metabolic water production may be insufficient to prevent dehydration, in honeybees. Thus, urban gardeners in any city may want to consider providing water sources for honeybees.

Finally, we note that although bumblebee hygric safety margins were not associated with urbanization, bumblebees had narrow hygric safety margins overall. This suggests that bumblebees are able to maintain their water content with increasing urbanization in this cool, mesic city, but urban bumblebees may be at greater risk in drier or hotter cities or during heatwaves or droughts.

### Conclusion

Here, we provide the first measurements of bee CWC and one of only a small number examining bee CT_max_ (only one other in an urban context). Interesting insights emerge from these investigations. First, we found that species differ in their thermal and desiccation tolerance and in their safety margins. Honeybees may be particularly sensitive to desiccation associated with urbanization and climate change, while sweat bees may be more sensitive to temperature changes. More research is needed to determine whether bumblebees are more sensitive to changes in temperature or water balance. This is because, for bumblebees, we found a decline in thermal safety margins, but not hygric safety margins, with urbanization, but overall hygric safety margins were much narrower than their thermal safety margins, indicating potential susceptibility to changes in water balance.

Overall, we argue that a physiological approach provides important information about how bees may respond to changes in climate and land use. Additionally, our research highlights that water balance should not be ignored. Moreover, if changes in bee thermal and water physiology associated with land use or climate change alters bee communities, there could be significant economic consequences related to altered pollination services. Thus, physiological approaches could prove useful in developing strategies to maintain key ecosystem services under climate and land use change.

## Supplementary information

Supplementary Information

## Data Availability

All data analyzed in this study will be made available in supplementary files.
