# Supplementary material for: Differential sensitivity of bees to urbanization-driven changes in body temperature and water content
Source: Sci Rep. 2019 Feb 7;9:1643. doi: 10.1038/s41598-018-38338-0 (PMC6367438; doi:10.1038/s41598-018-38338-0)
Supplement: Supplementary file 1 — Supplementary Information [file 41598_2018_38338_MOESM1_ESM.docx]

**Supplemental Information**

TITLE: Differential sensitivity of bees to urbanization-driven changes in body temperature and water content

AUTHORS: Justin D Burdine, Kevin E McCluney

**S1**. Graph displaying CT_max_ between site class (χ^2^ = 3.84, p > 0.05). The three investigated bee species at urban sites (52.39°C ± 0.77) and rural sites (49.57°C ± 8 1.13). Each data point represents an individual bee.


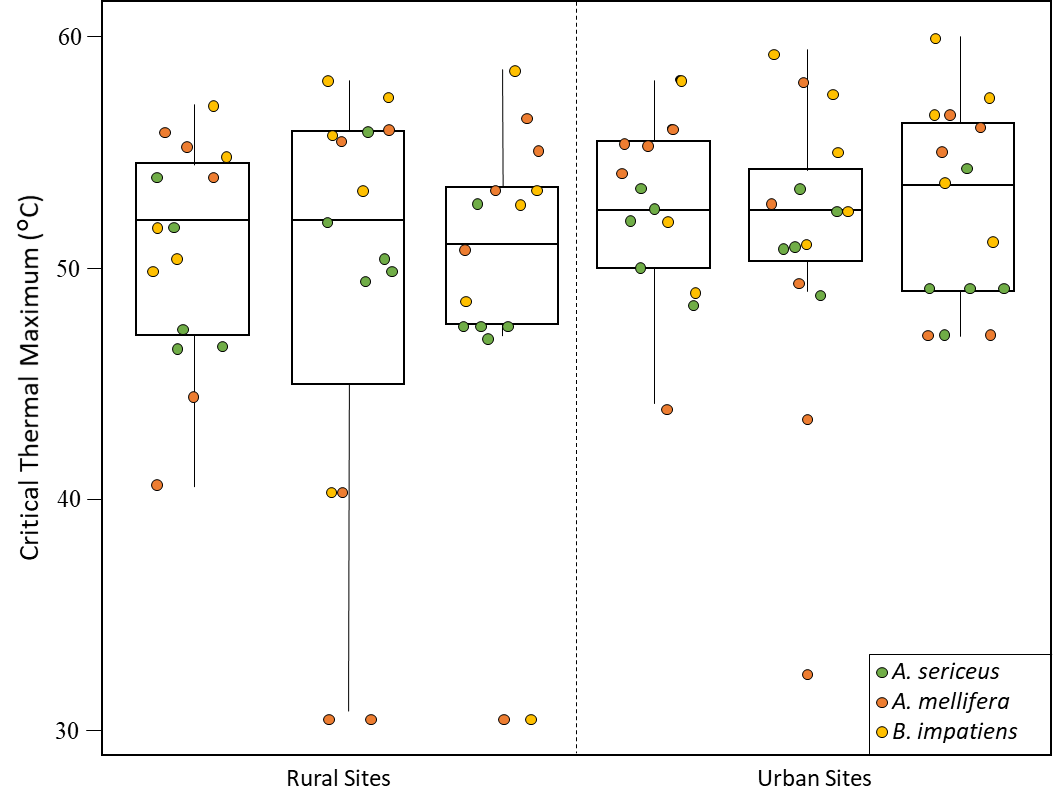


**S2**. Panel figure displaying the relationship between temperature (CT_max_ and Field Body Temperature) and percent imperviousness surface for the three investigated bee species. We show these relationships at the scale of (A) local (300 m) percent impervious surface, and (B) landscape (2000 m) percent impervious surface.


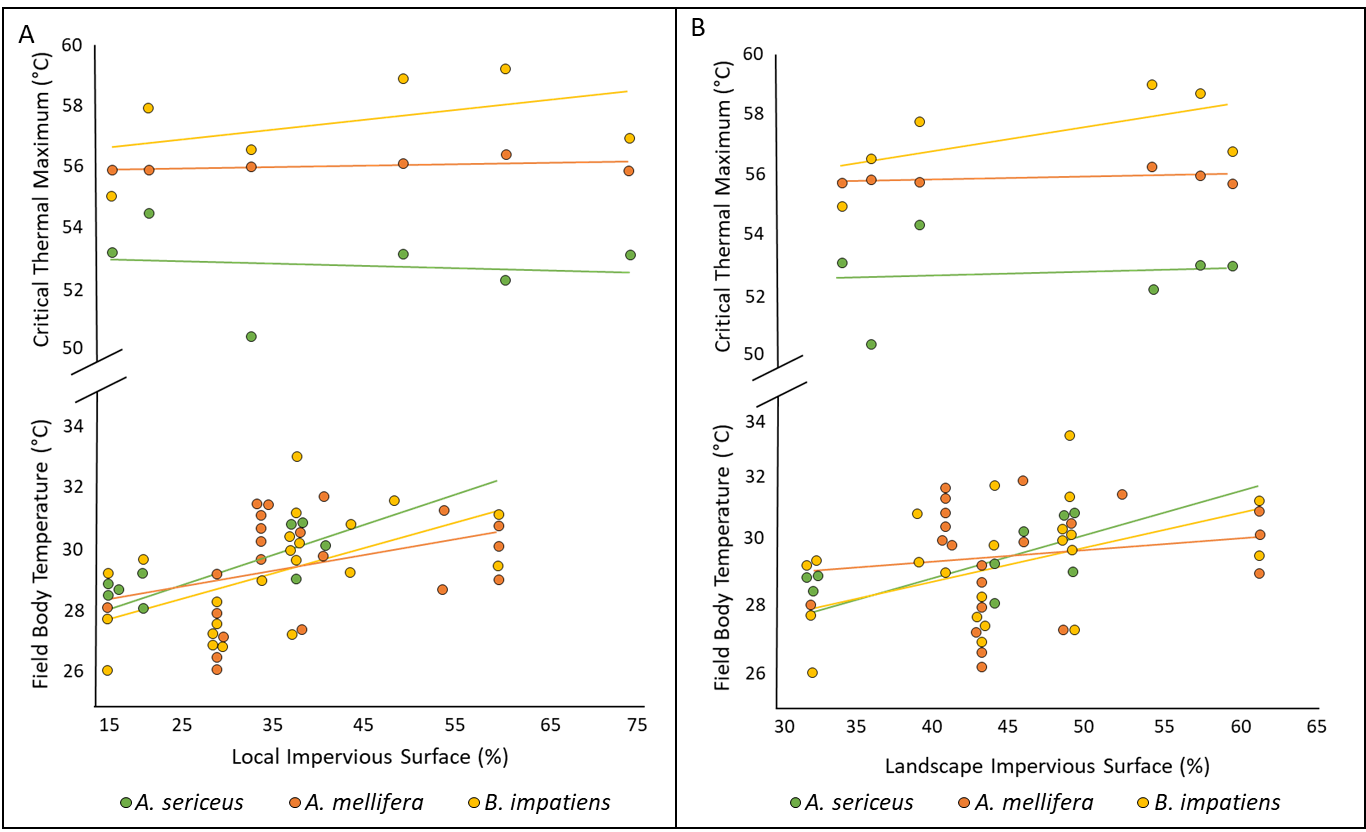


**S3**. Panel figure displaying the relationship for the three investigated bee species between local (300 m) percent impervious surface and (A) field body water content, and (B) critical water content.


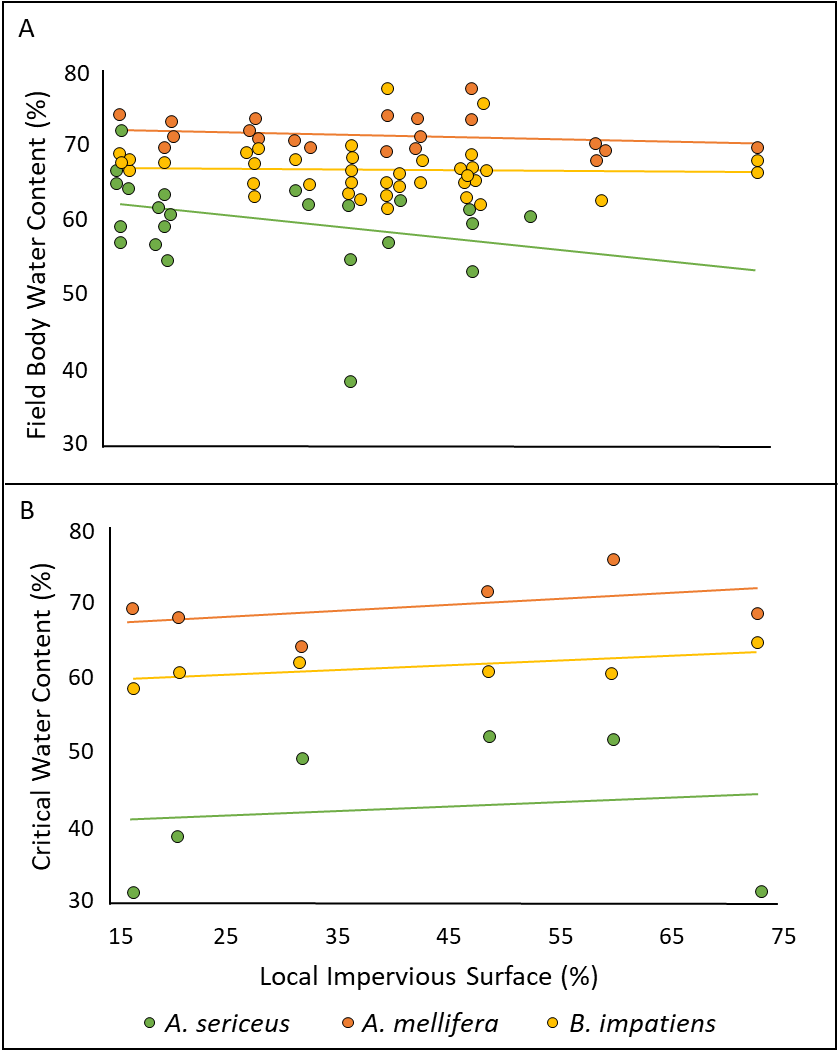


**S4**. Panel figure displaying the relationship for the three investigated bee species between landscape (2000 m) percent impervious surface and (A) field body water content, and (B) critical water content.


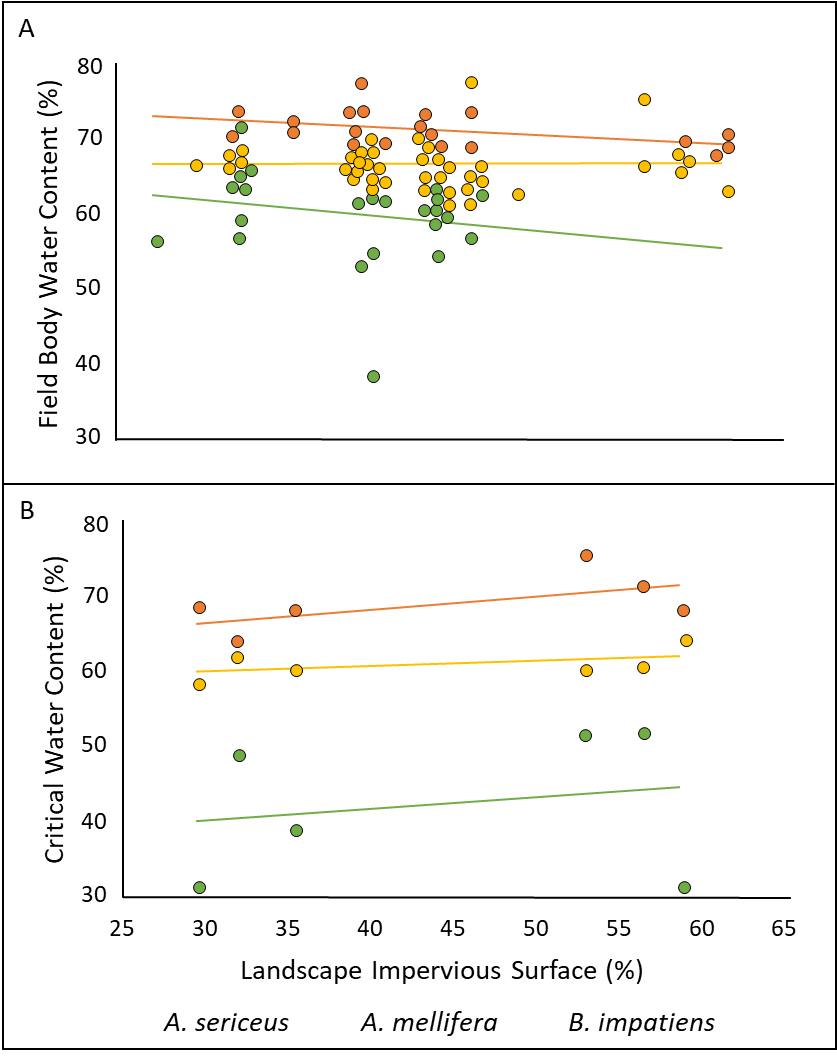


**S5**. Table displaying all data used for the calculation of critical thermal maximum (CTmax). The table displays site names (Site), site geographical location (Lat = latitude; Long=longitude), and classification (Class). The table also includes the mass (Mass) of each bee (in mg), species name (Species), and the recorded CTmax (in °C).

| **Record** | **Site** | **Lat** | **Long** | **Class** | **Mass** | **Species** | **CTmax** |
| --- | --- | --- | --- | --- | --- | --- | --- |
| 1 | MCD | 41.641 | -83.513 | Urban | 5.357 | Agapostemon sericeus | 54.5 |
| 2 | DOY | 41.681 | -83.524 | Urban | 29.554 | Bombus impatiens | 52.5 |
| 3 | CHE | 41.657 | -83.549 | Urban | 34.161 | Bombus impatiens | 49 |
| 4 | SYL | 41.696 | -83.752 | Rural | 45.836 | Bombus impatiens | 48.5 |
| 5 | BU | 41.548 | -83.671 | Rural | 18.905 | Apis mellifera | 40.5 |
| 6 | BU | 41.548 | -83.671 | Rural | 19.342 | Apis mellifera | 44.5 |
| 7 | CHE | 41.657 | -83.549 | Urban | 21.593 | Apis mellifera | 55.5 |
| 8 | SV | 41.682 | -83.717 | Rural | 19.382 | Apis mellifera | 40.5 |
| 9 | MCD | 41.641 | -83.513 | Urban | 23.622 | Apis mellifera | 56.5 |
| 10 | DOY | 41.681 | -83.524 | Urban | 55.572 | Bombus impatiens | 57.5 |
| 11 | MCD | 41.641 | -83.513 | Urban | 23.371 | Apis mellifera | 47 |
| 12 | SV | 41.682 | -83.717 | Rural | 18.749 | Apis mellifera | 56 |
| 13 | SV | 41.682 | -83.717 | Rural | 36.974 | Bombus impatiens | 56 |
| 14 | SYL | 41.696 | -83.752 | Rural | 18.858 | Apis mellifera | 53.5 |
| 15 | MCD | 41.641 | -83.513 | Urban | 39.483 | Bombus impatiens | 53.5 |
| 16 | DOY | 41.681 | -83.524 | Urban | 32.692 | Bombus impatiens | 55 |
| 17 | MCD | 41.641 | -83.513 | Urban | 25.104 | Apis mellifera | 56 |
| 18 | SYL | 41.696 | -83.752 | Rural | 22.023 | Apis mellifera | 56.5 |
| 19 | DOY | 41.681 | -83.524 | Urban | 50.401 | Bombus impatiens | 59.5 |
| 20 | BU | 41.548 | -83.671 | Rural | 24.836 | Apis mellifera | 56 |
| 21 | CHE | 41.657 | -83.549 | Urban | 23.619 | Apis mellifera | 44 |
| 22 | SV | 41.682 | -83.717 | Rural | 38.181 | Bombus impatiens | 57.5 |
| 23 | BU | 41.548 | -83.671 | Rural | 21.565 | Apis mellifera | 54 |
| 24 | SYL | 41.696 | -83.752 | Rural | 20.276 | Apis mellifera | 55 |
| 25 | SYL | 41.696 | -83.752 | Rural | 52.24 | Bombus impatiens | 53 |
| 26 | BU | 41.548 | -83.671 | Rural | 35.834 | Bombus impatiens | 50 |
| 27 | SV | 41.682 | -83.717 | Rural | 17.35 | Apis mellifera | 30.5 |
| 28 | MCD | 41.641 | -83.513 | Urban | 24.865 | Apis mellifera | 55 |
| 29 | BU | 41.548 | -83.671 | Rural | 44.017 | Bombus impatiens | 50.5 |
| 30 | BU | 41.548 | -83.671 | Rural | 40.947 | Bombus impatiens | 52 |
| 31 | MCD | 41.641 | -83.513 | Urban | 7.891 | Agapostemon sericeus | 49 |
| 32 | SYL | 41.696 | -83.752 | Rural | 60.597 | Bombus impatiens | 30.5 |
| 33 | SV | 41.682 | -83.717 | Rural | 44.457 | Bombus impatiens | 53.5 |
| 34 | SV | 41.682 | -83.717 | Rural | 23.211 | Apis mellifera | 55.5 |
| 35 | SYL | 41.696 | -83.752 | Rural | 17.328 | Apis mellifera | 30.5 |
| 36 | SV | 41.682 | -83.717 | Rural | 17.907 | Apis mellifera | 30.5 |
| 37 | DOY | 41.681 | -83.524 | Urban | 38.585 | Bombus impatiens | 51 |
| 38 | SYL | 41.696 | -83.752 | Rural | 54.37 | Bombus impatiens | 58.5 |
| 39 | SV | 41.682 | -83.717 | Rural | 33.609 | Bombus impatiens | 40.5 |
| 40 | SYL | 41.696 | -83.752 | Rural | 19.036 | Apis mellifera | 51 |
| 41 | BU | 41.548 | -83.671 | Rural | 49.561 | Bombus impatiens | 55 |
| 42 | CHE | 41.657 | -83.549 | Urban | 18.874 | Apis mellifera | 56 |
| 43 | BU | 41.548 | -83.671 | Rural | 25.751 | Bombus impatiens | 57 |
| 44 | MCD | 41.641 | -83.513 | Urban | 25.367 | Apis mellifera | 47 |
| 45 | MCD | 41.641 | -83.513 | Urban | 24.608 | Bombus impatiens | 56.5 |
| 46 | BU | 41.548 | -83.671 | Rural | 21.604 | Apis mellifera | 55.5 |
| 47 | MCD | 41.641 | -83.513 | Urban | 99.73 | Bombus impatiens | 51 |
| 48 | SYL | 41.696 | -83.752 | Rural | 65.474 | Bombus impatiens | 53.5 |
| 49 | MCD | 41.641 | -83.513 | Urban | 35.3 | Bombus impatiens | 57.5 |
| 50 | CHE | 41.657 | -83.549 | Urban | 47.678 | Bombus impatiens | 52 |
| 51 | CHE | 41.657 | -83.549 | Urban | 22.892 | Apis mellifera | 55.5 |
| 52 | CHE | 41.657 | -83.549 | Urban | 56.026 | Bombus impatiens | 58 |
| 53 | SV | 41.682 | -83.717 | Rural | 38.147 | Bombus impatiens | 58 |
| 54 | CHE | 41.657 | -83.549 | Urban | 19.78 | Apis mellifera | 54 |
| 55 | MCD | 41.641 | -83.513 | Urban | 1.057 | Agapostemon sericeus | 49 |
| 56 | SYL | 41.696 | -83.752 | Rural | 2.388 | Agapostemon sericeus | 47 |
| 57 | SYL | 41.696 | -83.752 | Rural | 4.081 | Agapostemon sericeus | 47.5 |
| 58 | SV | 41.682 | -83.717 | Rural | 4.863 | Agapostemon sericeus | 50 |
| 59 | CHE | 41.657 | -83.549 | Urban | 4.2 | Agapostemon sericeus | 48.5 |
| 60 | SYL | 41.696 | -83.752 | Rural | 2.552 | Agapostemon sericeus | 47.5 |
| 61 | BU | 41.548 | -83.671 | Rural | 1.384 | Agapostemon sericeus | 46.5 |
| 62 | SV | 41.682 | -83.717 | Rural | 2 | Agapostemon sericeus | 49.5 |
| 63 | SYL | 41.696 | -83.752 | Rural | 2.053 | Agapostemon sericeus | 47.5 |
| 64 | CHE | 41.657 | -83.549 | Urban | 7.078 | Agapostemon sericeus | 52 |
| 65 | BU | 41.548 | -83.671 | Rural | 2.599 | Agapostemon sericeus | 54 |
| 66 | SV | 41.682 | -83.717 | Rural | 8.731 | Agapostemon sericeus | 56 |
| 67 | CHE | 41.657 | -83.549 | Urban | 6.705 | Agapostemon sericeus | 52.5 |
| 68 | SV | 41.682 | -83.717 | Rural | 3.819 | Agapostemon sericeus | 50.5 |
| 69 | CHE | 41.657 | -83.549 | Urban | 4.637 | Agapostemon sericeus | 50 |
| 70 | BU | 41.548 | -83.671 | Rural | 6.89 | Agapostemon sericeus | 47.5 |
| 71 | BU | 41.548 | -83.671 | Rural | 5.281 | Agapostemon sericeus | 52 |
| 72 | DOY | 41.681 | -83.524 | Urban | 1.141 | Agapostemon sericeus | 53.5 |
| 73 | DOY | 41.681 | -83.524 | Urban | 1.91 | Agapostemon sericeus | 49 |
| 74 | DOY | 41.681 | -83.524 | Urban | 1.896 | Agapostemon sericeus | 51 |
| 75 | SYL | 41.696 | -83.752 | Rural | 2.69 | Agapostemon sericeus | 52.5 |
| 76 | SV | 41.682 | -83.717 | Rural | 7.976 | Agapostemon sericeus | 52 |
| 77 | MCD | 41.641 | -83.513 | Urban | 8.384 | Agapostemon sericeus | 47 |
| 78 | BU | 41.548 | -83.671 | Rural | 3.915 | Agapostemon sericeus | 46.5 |
| 79 | MCD | 41.641 | -83.513 | Urban | 1.48 | Agapostemon sericeus | 49 |
| 80 | DOY | 41.681 | -83.524 | Urban | 22.852 | Apis mellifera | 49.5 |
| 81 | DOY | 41.681 | -83.524 | Urban | 25.399 | Apis mellifera | 43.5 |
| 82 | DOY | 41.681 | -83.524 | Urban | 20.537 | Apis mellifera | 53 |
| 83 | DOY | 41.681 | -83.524 | Urban | 19.981 | Apis mellifera | 58 |
| 84 | DOY | 41.681 | -83.524 | Urban | 23.449 | Apis mellifera | 32.5 |
| 85 | DOY | 41.681 | -83.524 | Urban | 2.749 | Agapostemon sericeus | 52.5 |
| 86 | DOY | 41.681 | -83.524 | Urban | 1.865 | Agapostemon sericeus | 51 |
| 87 | MCD | 41.641 | -83.513 | Urban | 49.251 | Bombus impatiens | 60 |
| 88 | CHE | 41.657 | -83.549 | Urban | 2.355 | Agapostemon sericeus | 53.5 |

**S6**. Table displaying all data used for the calculation of critical water content (CWC). The table displays site names (Site), geographical locations (Lat = latitude; Long=longitude), and classification (Class). The table also includes the mass (Mass) of each bee (in mg), species name (Species), desiccation survival time in hours (Time), and CWC.

| **Record** | **Site** | **Lat** | **Long** | **Class** | **Mass** | **Species** | **Time** | **CWC** |
| --- | --- | --- | --- | --- | --- | --- | --- | --- |
| 1 | DOY | 41.681 | -83.524 | Urban | 39.756 | Bombus impatiens | 23 | 65.348 |
| 2 | MCD | 41.641 | -83.513 | Urban | 45.306 | Bombus impatiens | 32 | 58.953 |
| 3 | SV | 41.682 | -83.717 | Rural | 34.277 | Bombus impatiens | 41 | 63.718 |
| 4 | SV | 41.682 | -83.717 | Rural | 37.028 | Bombus impatiens | 8 | 60.647 |
| 5 | BU | 41.548 | -83.671 | Rural | 21.421 | Apis mellifera | 8 | 71.314 |
| 6 | BU | 41.548 | -83.671 | Rural | 11.555 | Agapostemon sericeus | 8 | 35.262 |
| 7 | MCD | 41.641 | -83.513 | Urban | 20.046 | Apis mellifera | 9 | 73.636 |
| 8 | SV | 41.682 | -83.717 | Rural | 6.028 | Agapostemon sericeus | 8 | 33.119 |
| 9 | BU | 41.548 | -83.671 | Rural | 21.456 | Apis mellifera | 3 | 70.375 |
| 10 | CHE | 41.657 | -83.549 | Urban | 1.731 | Agapostemon sericeus | 17 | 48.065 |
| 11 | CHE | 41.657 | -83.549 | Urban | 21.161 | Apis mellifera | 0.5 | 68.021 |
| 12 | BU | 41.548 | -83.671 | Rural | 2.862 | Agapostemon sericeus | 8 | 33.750 |
| 13 | BU | 41.548 | -83.671 | Rural | 31.701 | Bombus impatiens | 23 | 61.214 |
| 14 | CHE | 41.657 | -83.549 | Urban | 6.439 | Agapostemon sericeus | 8 | 53.003 |
| 15 | BU | 41.548 | -83.671 | Rural | 20.475 | Apis mellifera | 8 | 67.889 |
| 16 | DOY | 41.681 | -83.524 | Urban | 32.048 | Bombus impatiens | 23 | 60.384 |
| 17 | MCD | 41.641 | -83.513 | Urban | 0.364 | Agapostemon sericeus | 23 | 72.937 |
| 18 | BU | 41.548 | -83.671 | Rural | 38.442 | Bombus impatiens | 23 | 57.384 |
| 19 | CHE | 41.657 | -83.549 | Urban | 0.962 | Agapostemon sericeus | 17 | 21.277 |
| 20 | SV | 41.682 | -83.717 | Rural | 41.514 | Bombus impatiens | 17 | 60.319 |
| 21 | MCD | 41.641 | -83.513 | Urban | 22.729 | Apis mellifera | 9 | 72.759 |
| 22 | CHE | 41.657 | -83.549 | Urban | 18.207 | Apis mellifera | 8 | 76.906 |
| 23 | SYL | 41.696 | -83.752 | Rural | 51.277 | Bombus impatiens | 8 | 64.622 |
| 24 | DOY | 41.681 | -83.524 | Urban | 2.319 | Agapostemon sericeus | 17 | 64.943 |
| 25 | SV | 41.682 | -83.717 | Rural | 31.339 | Bombus impatiens | 3 | 60.699 |
| 26 | SV | 41.682 | -83.717 | Rural | 20.531 | Apis mellifera | 210 | 82.654 |
| 27 | BU | 41.548 | -83.671 | Rural | 20.062 | Apis mellifera | 2 | 77.912 |
| 28 | SYL | 41.696 | -83.752 | Rural | 51.618 | Bombus impatiens | 17 | 61.031 |
| 29 | SYL | 41.696 | -83.752 | Rural | 7.329 | Agapostemon sericeus | 8 | 52.111 |
| 30 | DOY | 41.681 | -83.524 | Urban | 34.945 | Bombus impatiens | 9 | 63.174 |
| 31 | SYL | 41.696 | -83.752 | Rural | 20.092 | Apis mellifera | 3 | 81.695 |
| 32 | DOY | 41.681 | -83.524 | Urban | 20.495 | Apis mellifera | 9 | 74.436 |
| 33 | MCD | 41.641 | -83.513 | Urban | 21.79 | Apis mellifera | 1 | 70.007 |
| 34 | BU | 41.548 | -83.671 | Rural | 4.398 | Agapostemon sericeus | 8 | 29.778 |
| 35 | DOY | 41.681 | -83.524 | Urban | 6.815 | Agapostemon sericeus | 9 | 48.017 |
| 36 | BU | 41.548 | -83.671 | Rural | 30.782 | Bombus impatiens | 17 | 63.770 |
| 37 | MCD | 41.641 | -83.513 | Urban | 35.172 | Bombus impatiens | 9 | 63.651 |
| 38 | DOY | 41.681 | -83.524 | Urban | 30.05 | Bombus impatiens | 32 | 65.476 |
| 39 | DOY | 41.681 | -83.524 | Urban | 20.527 | Apis mellifera | 9 | 81.231 |
| 40 | BU | 41.548 | -83.671 | Rural | 21.377 | Apis mellifera | 8 | 69.389 |
| 41 | SYL | 41.696 | -83.752 | Rural | 31.193 | Bombus impatiens | 8 | 65.085 |
| 42 | SYL | 41.696 | -83.752 | Rural | 31.582 | Bombus impatiens | 17 | 64.932 |
| 43 | CHE | 41.657 | -83.549 | Urban | 7.452 | Agapostemon sericeus | 17 | 46.218 |
| 44 | DOY | 41.681 | -83.524 | Urban | 21.905 | Apis mellifera | 9 | 76.695 |
| 45 | DOY | 41.681 | -83.524 | Urban | 20.492 | Apis mellifera | 9 | 79.572 |
| 46 | BU | 41.548 | -83.671 | Rural | 4.488 | Agapostemon sericeus | 17 | 37.240 |
| 47 | BU | 41.548 | -83.671 | Rural | 41.181 | Bombus impatiens | 23 | 64.920 |
| 48 | DOY | 41.681 | -83.524 | Urban | 18.175 | Apis mellifera | 9 | 82.232 |
| 49 | SYL | 41.696 | -83.752 | Rural | 22.404 | Apis mellifera | 2 | 66.118 |
| 50 | SV | 41.682 | -83.717 | Rural | 18.49 | Apis mellifera | 0.25 | 67.969 |
| 51 | SV | 41.682 | -83.717 | Rural | 17.414 | Apis mellifera | 0.25 | 71.124 |
| 52 | SV | 41.682 | -83.717 | Rural | 16.271 | Apis mellifera | 0.25 | 67.600 |
| 53 | SYL | 41.696 | -83.752 | Rural | 44.559 | Bombus impatiens | 0.5 | 64.309 |
| 54 | SYL | 41.696 | -83.752 | Rural | 21.254 | Apis mellifera | 8 | 82.038 |
| 55 | SYL | 41.696 | -83.752 | Rural | 21.05 | Apis mellifera | 1.5 | 76.566 |
| 56 | DOY | 41.681 | -83.524 | Urban | 32.866 | Bombus impatiens | 41 | 60.817 |
| 57 | SV | 41.682 | -83.717 | Rural | 19.046 | Apis mellifera | 2 | 71.350 |
| 58 | SYL | 41.696 | -83.752 | Rural | 19.264 | Apis mellifera | 3 | 62.137 |
| 59 | SYL | 41.696 | -83.752 | Rural | 5.78 | Agapostemon sericeus | 2 | 48.259 |
| 60 | SV | 41.682 | -83.717 | Rural | 5.297 | Agapostemon sericeus | 8 | 48.473 |
| 61 | SV | 41.682 | -83.717 | Rural | 0.846 | Agapostemon sericeus | 3 | 77.824 |
| 62 | SV | 41.682 | -83.717 | Rural | 5.332 | Agapostemon sericeus | 1.5 | 53.199 |
| 63 | DOY | 41.681 | -83.524 | Urban | 6.577 | Agapostemon sericeus | 95 | 69.513 |
| 64 | MCD | 41.641 | -83.513 | Urban | 3.746 | Agapostemon sericeus | 95 | 53.338 |
| 65 | MCD | 41.641 | -83.513 | Urban | 2.137 | Agapostemon sericeus | 95 | 57.825 |
| 66 | MCD | 41.641 | -83.513 | Urban | 1.068 | Agapostemon sericeus | 95 | 51.322 |
| 67 | SV | 41.682 | -83.717 | Rural | 4.541 | Agapostemon sericeus | 168 | 50.690 |
| 68 | BU | 41.548 | -83.671 | Rural | 8.958 | Agapostemon sericeus | 9 | 56.426 |
| 69 | CHE | 41.657 | -83.549 | Urban | 6.055 | Agapostemon sericeus | 48 | 64.384 |
| 70 | MCD | 41.641 | -83.513 | Urban | 7.271 | Agapostemon sericeus | 48 | 60.220 |
| 71 | DOY | 41.681 | -83.524 | Urban | 5.101 | Agapostemon sericeus | 72 | 63.866 |
| 72 | DOY | 41.681 | -83.524 | Urban | 10.894 | Agapostemon sericeus | 96 | 57.260 |
| 73 | SYL | 41.696 | -83.752 | Rural | 2.41 | Agapostemon sericeus | 17 | 57.186 |
| 74 | SYL | 41.696 | -83.752 | Rural | 5.944 | Agapostemon sericeus | 48 | 50.343 |
| 75 | SYL | 41.696 | -83.752 | Rural | 5.065 | Agapostemon sericeus | 120 | 53.841 |
| 76 | CHE | 41.657 | -83.549 | Urban | 18.76 | Apis mellifera | 2 | 68.596 |
| 77 | CHE | 41.657 | -83.549 | Urban | 18.779 | Apis mellifera | 9 | 79.758 |
| 78 | CHE | 41.657 | -83.549 | Urban | 19.355 | Apis mellifera | 9 | 77.230 |
| 79 | MCD | 41.641 | -83.513 | Urban | 21.001 | Apis mellifera | 9 | 78.046 |
| 80 | MCD | 41.641 | -83.513 | Urban | 20.246 | Apis mellifera | 9 | 80.826 |
| 81 | CHE | 41.657 | -83.549 | Urban | 31.175 | Bombus impatiens | 9 | 64.186 |
| 82 | CHE | 41.657 | -83.549 | Urban | 34.536 | Bombus impatiens | 9 | 66.482 |
| 83 | CHE | 41.657 | -83.549 | Urban | 29.883 | Bombus impatiens | 17 | 67.037 |
| 84 | CHE | 41.657 | -83.549 | Urban | 36.577 | Bombus impatiens | 72 | 64.723 |
| 85 | CHE | 41.657 | -83.549 | Urban | 38.48 | Bombus impatiens | 23 | 67.939 |
| 86 | MCD | 41.641 | -83.513 | Urban | 58.373 | Bombus impatiens | 17 | 66.656 |
| 87 | MCD | 41.641 | -83.513 | Urban | 42.144 | Bombus impatiens | 17 | 64.755 |
| 88 | MCD | 41.641 | -83.513 | Urban | 55.384 | Bombus impatiens | 9 | 65.408 |
| 89 | SV | 41.682 | -83.717 | Rural | 31.558 | Bombus impatiens | 17 | 68.329 |

**S7**. Table displaying all data used for the calculation of thermal safety margin (TSM). The table displays site names (Site), geographical locations (Lat = latitude; Long=longitude), and measurements of local (Local: 300m) and landscape (Landscape: 2000m) percent impervious surface. The table also includes the mass (Mass) of each bee (in mg), species name (Species), field body temperatures (Temp: in °C), and TSM (in °C).

| **Record** | **Site** | **Lat** | **Long** | **Local** | **Landscape** | **Mass** | **Species** | **Temp** | **TSM** |
| --- | --- | --- | --- | --- | --- | --- | --- | --- | --- |
| 1 | TV | 41.607 | -83.657 | 36.93 | 40.05 | 32.793 | Bombus impatiens | 27.3 | 31.3 |
| 2 | TV | 41.607 | -83.657 | 36.93 | 40.05 | 32.455 | Bombus impatiens | 29.7 | 28.9 |
| 3 | TV | 41.607 | -83.657 | 36.93 | 40.05 | 26.512 | Bombus impatiens | 30 | 28.6 |
| 4 | TV | 41.607 | -83.657 | 36.93 | 40.05 | 44.711 | Bombus impatiens | 30.2 | 28.4 |
| 5 | TUC | 41.637 | -83.654 | 33.14 | 40.85 | 20.823 | Apis mellifera | 31.2 | 25.25 |
| 6 | TV | 41.607 | -83.657 | 36.93 | 40.05 | 59.409 | Bombus impatiens | 33 | 25.6 |
| 7 | TUC | 41.637 | -83.654 | 33.14 | 40.85 | 59.379 | Bombus impatiens | 29 | 29.6 |
| 8 | MNR | 41.670 | -83.584 | 40.10 | 45.92 | 22.457 | Apis mellifera | 29.9 | 26.55 |
| 9 | TV | 41.607 | -83.657 | 36.93 | 40.05 | 54.005 | Bombus impatiens | 31.2 | 27.4 |
| 10 | TV | 41.607 | -83.657 | 36.93 | 40.05 | 4.448 | Agapostemon sericeus | 30.7 | 23.25 |
| 11 | ZL | 41.621 | -83.585 | 47.74 | 44.26 | 66.098 | Bombus impatiens | 31.6 | 27 |
| 12 | TV | 41.607 | -83.657 | 36.93 | 40.05 | 4.427 | Agapostemon sericeus | 29 | 24.95 |
| 13 | TV | 41.607 | -83.657 | 36.93 | 40.05 | 5.347 | Agapostemon sericeus | 30.6 | 23.35 |
| 14 | TV | 41.607 | -83.657 | 36.93 | 40.05 | 46.064 | Bombus impatiens | 30.4 | 28.2 |
| 15 | TV | 41.607 | -83.657 | 36.93 | 40.05 | 34.796 | Apis mellifera | 30.6 | 25.85 |
| 16 | OWN | 41.584 | -83.541 | 53.19 | 52.55 | 25.635 | Apis mellifera | 31.3 | 25.15 |
| 17 | INT | 41.647 | -83.527 | 59.28 | 61.33 | 31.604 | Apis mellifera | 30.9 | 25.55 |
| 18 | FX | 41.699 | -83.628 | 28.28 | 43.28 | 40.832 | Bombus impatiens | 28.3 | 30.3 |
| 19 | INT | 41.647 | -83.527 | 59.28 | 61.33 | 53.571 | Bombus impatiens | 29.44 | 29.16 |
| 20 | MNR | 41.670 | -83.584 | 40.10 | 45.92 | 25.44 | Apis mellifera | 31.7 | 24.75 |
| 21 | FX | 41.699 | -83.628 | 28.28 | 43.28 | 25.552 | Apis mellifera | 26.2 | 30.25 |
| 22 | UT | 41.659 | -83.621 | 42.99 | 39.05 | 27.337 | Bombus impatiens | 30.8 | 27.8 |
| 23 | FX | 41.699 | -83.628 | 28.28 | 43.28 | 28.687 | Bombus impatiens | 26.9 | 31.7 |
| 24 | FX | 41.699 | -83.628 | 28.28 | 43.28 | 27.506 | Bombus impatiens | 27.4 | 31.2 |
| 25 | INT | 41.647 | -83.527 | 59.28 | 61.33 | 24.155 | Apis mellifera | 29 | 27.45 |
| 26 | INT | 41.647 | -83.527 | 59.28 | 61.33 | 20.729 | Apis mellifera | 30.1 | 26.35 |
| 27 | CC | 41.591 | -83.441 | 16.24 | 32.16 | 31.871 | Apis mellifera | 28.1 | 28.35 |
| 28 | UT | 41.659 | -83.621 | 42.99 | 39.05 | 54.764 | Bombus impatiens | 29.3 | 29.3 |
| 29 | INT | 41.647 | -83.527 | 59.28 | 61.33 | 34.325 | Bombus impatiens | 31 | 27.6 |
| 30 | CC | 41.591 | -83.441 | 16.24 | 32.16 | 77.09 | Bombus impatiens | 27.8 | 30.8 |
| 31 | FX | 41.699 | -83.628 | 28.28 | 43.28 | 24.895 | Apis mellifera | 27.9 | 28.55 |
| 32 | FX | 41.699 | -83.628 | 28.28 | 43.28 | 22.077 | Apis mellifera | 27.3 | 29.15 |
| 33 | FX | 41.699 | -83.628 | 28.28 | 43.28 | 27 | Bombus impatiens | 27.7 | 30.9 |
| 34 | FX | 41.699 | -83.628 | 28.28 | 43.28 | 22.132 | Bombus impatiens | 26.9 | 31.7 |
| 35 | FX | 41.699 | -83.628 | 28.28 | 43.28 | 22.589 | Apis mellifera | 29.2 | 27.25 |
| 36 | FX | 41.699 | -83.628 | 28.28 | 43.28 | 34.476 | Apis mellifera | 26.6 | 29.85 |
| 37 | TUC | 41.637 | -83.654 | 33.14 | 40.85 | 27.069 | Apis mellifera | 30.3 | 26.15 |
| 38 | TUC | 41.637 | -83.654 | 33.14 | 40.85 | 26.134 | Apis mellifera | 29.7 | 26.75 |
| 39 | TUC | 41.637 | -83.654 | 33.14 | 40.85 | 28.299 | Apis mellifera | 31.4 | 25.05 |
| 40 | TUC | 41.637 | -83.654 | 33.14 | 40.85 | 24.915 | Apis mellifera | 31.4 | 25.05 |
| 41 | CC | 41.591 | -83.441 | 16.24 | 32.16 | 27.523 | Bombus impatiens | 26.1 | 32.5 |
| 42 | CC | 41.591 | -83.441 | 16.24 | 32.16 | 34.814 | Bombus impatiens | 29.1 | 29.5 |
| 43 | CHS | 41.684 | -83.496 | 37.03 | 48.87 | 21.592 | Apis mellifera | 27.3 | 29.15 |
| 44 | TUC | 41.637 | -83.654 | 33.14 | 40.85 | 23.84 | Apis mellifera | 30.7 | 25.75 |
| 45 | COL | 41.663 | -83.483 | 20.24 | 44.06 | 46.352 | Bombus impatiens | 29.7 | 28.9 |
| 46 | CC | 41.591 | -83.441 | 16.24 | 32.16 | 5.962 | Agapostemon sericeus | 29 | 24.95 |
| 47 | ZP | 41.618 | -83.578 | 53.06 | 43.30 | 17.094 | Apis mellifera | 28.7 | 27.75 |
| 48 | MNR | 41.670 | -83.584 | 40.10 | 45.92 | 6.73 | Agapostemon sericeus | 30.1 | 23.85 |
| 49 | COL | 41.663 | -83.483 | 20.24 | 44.06 | 5.247 | Agapostemon sericeus | 29.3 | 24.65 |
| 50 | COL | 41.663 | -83.483 | 20.24 | 44.06 | 10.287 | Agapostemon sericeus | 28.1 | 25.85 |
| 51 | CC | 41.591 | -83.441 | 16.24 | 32.16 | 2.789 | Agapostemon sericeus | 26.1 | 27.85 |
| 52 | CC | 41.591 | -83.441 | 16.24 | 32.16 | 6.249 | Agapostemon sericeus | 28.5 | 25.45 |

**S8**. Table displaying all data used for the calculation of hygric safety margin (HSM). The table displays site names (Site), geographical locations (Lat = latitude; Long=longitude), and measurements of local (Local: 300m) and landscape (Landscape: 2000m) percent impervious surface. The table also includes the mass (Mass) of each bee (in mg), species name (Species), and HSM (%).

| **Record** | **Site** | **Lat** | **Long** | **Local** | **Landscape** | **Mass** | **Species** | **HSM** |
| --- | --- | --- | --- | --- | --- | --- | --- | --- |
| 1 | TV | 41.607 | -83.657 | 36.935 | 40.055 | 27.586 | Bombus impatiens | 5.69 |
| 2 | SV | 41.682 | -83.717 | 20.878 | 35.425 | 19.13 | Apis mellifera | 2.55 |
| 3 | TV | 41.607 | -83.657 | 36.935 | 40.055 | 32.793 | Bombus impatiens | 7.18 |
| 4 | TV | 41.607 | -83.657 | 36.935 | 40.055 | 32.455 | Bombus impatiens | 8.24 |
| 5 | FTU | 41.611 | -83.619 | 47.690 | 39.347 | 40.281 | Bombus impatiens | 4.91 |
| 6 | FTU | 41.611 | -83.619 | 47.690 | 39.347 | 45.233 | Bombus impatiens | 7.15 |
| 7 | CHE | 41.657 | -83.549 | 73.297 | 58.649 | 37.813 | Bombus impatiens | 6.81 |
| 8 | FTU | 41.611 | -83.619 | 47.690 | 39.347 | 40.262 | Bombus impatiens | 5.82 |
| 9 | FTU | 41.611 | -83.619 | 47.690 | 39.347 | 43.961 | Bombus impatiens | 5.94 |
| 10 | SYL | 41.696 | -83.752 | 32.000 | 31.829 | 5.754 | Agapostemon sericeus | 30.04 |
| 11 | SYL | 41.696 | -83.752 | 32.000 | 31.829 | 90.22 | Bombus impatiens | 6.54 |
| 12 | SYL | 41.696 | -83.752 | 32.000 | 31.829 | 26.119 | Apis mellifera | 1.90 |
| 13 | SV | 41.682 | -83.717 | 20.878 | 35.425 | 21.034 | Apis mellifera | 3.92 |
| 14 | DOY | 41.681 | -83.524 | 48.658 | 56.269 | 70.387 | Bombus impatiens | 5.32 |
| 15 | DOY | 41.681 | -83.524 | 48.658 | 56.269 | 62.51 | Bombus impatiens | 14.10 |
| 16 | CHS | 41.684 | -83.496 | 37.025 | 48.875 | 28.153 | Bombus impatiens | 1.89 |
| 17 | TV | 41.607 | -83.657 | 36.935 | 40.055 | 26.512 | Bombus impatiens | 3.51 |
| 18 | TV | 41.607 | -83.657 | 36.935 | 40.055 | 44.711 | Bombus impatiens | 2.44 |
| 19 | ZL | 41.621 | -83.585 | 47.741 | 44.260 | 74.237 | Bombus impatiens | 4.69 |
| 20 | MNR | 41.670 | -83.584 | 40.104 | 45.924 | 66.921 | Bombus impatiens | 2.74 |
| 21 | COL | 41.663 | -83.483 | 20.238 | 44.064 | 20.003 | Apis mellifera | 0.58 |
| 22 | ZL | 41.621 | -83.585 | 47.741 | 44.260 | 48.979 | Bombus impatiens | 1.70 |
| 23 | TV | 41.607 | -83.657 | 36.935 | 40.055 | 59.409 | Bombus impatiens | 5.37 |
| 24 | TUC | 41.637 | -83.654 | 33.143 | 40.848 | 59.379 | Bombus impatiens | 3.32 |
| 25 | MNR | 41.670 | -83.584 | 40.104 | 45.924 | 31.452 | Bombus impatiens | 0.45 |
| 26 | ZL | 41.621 | -83.585 | 47.741 | 44.260 | 14.045 | Agapostemon sericeus | 25.88 |
| 27 | FTU | 41.611 | -83.619 | 47.690 | 39.347 | 31.978 | Apis mellifera | 4.79 |
| 28 | TV | 41.607 | -83.657 | 36.935 | 40.055 | 4.448 | Agapostemon sericeus | 28.37 |
| 29 | ZL | 41.621 | -83.585 | 47.741 | 44.260 | 66.098 | Bombus impatiens | 0.93 |
| 30 | TV | 41.607 | -83.657 | 36.935 | 40.055 | 4.427 | Agapostemon sericeus | 5.16 |
| 31 | TV | 41.607 | -83.657 | 36.935 | 40.055 | 5.347 | Agapostemon sericeus | 21.45 |
| 32 | TV | 41.607 | -83.657 | 36.935 | 40.055 | 46.064 | Bombus impatiens | 4.26 |
| 33 | FTU | 41.611 | -83.619 | 47.690 | 39.347 | 25.814 | Apis mellifera | 8.57 |
| 34 | FX | 41.699 | -83.628 | 28.283 | 43.279 | 40.832 | Bombus impatiens | 2.40 |
| 35 | ZL | 41.621 | -83.585 | 47.741 | 44.260 | 56.855 | Bombus impatiens | 4.08 |
| 36 | FX | 41.699 | -83.628 | 28.283 | 43.279 | 25.552 | Apis mellifera | 2.14 |
| 37 | UT | 41.659 | -83.621 | 42.991 | 39.053 | 27.337 | Bombus impatiens | 6.74 |
| 38 | FX | 41.699 | -83.628 | 28.283 | 43.279 | 28.687 | Bombus impatiens | 8.38 |
| 39 | FX | 41.699 | -83.628 | 28.283 | 43.279 | 27.506 | Bombus impatiens | 7.86 |
| 40 | INT | 41.647 | -83.527 | 59.284 | 61.328 | 24.155 | Apis mellifera | 0.61 |
| 41 | INT | 41.647 | -83.527 | 59.284 | 61.328 | 20.729 | Apis mellifera | 1.33 |
| 42 | FTU | 41.611 | -83.619 | 47.690 | 39.347 | 4.535 | Agapostemon sericeus | 19.49 |
| 43 | CC | 41.591 | -83.441 | 16.245 | 32.162 | 31.871 | Apis mellifera | 4.83 |
| 44 | UT | 41.659 | -83.621 | 42.991 | 39.053 | 54.764 | Bombus impatiens | 3.97 |
| 45 | INT | 41.647 | -83.527 | 59.284 | 61.328 | 34.325 | Bombus impatiens | 1.62 |
| 46 | CC | 41.591 | -83.441 | 16.245 | 32.162 | 77.09 | Bombus impatiens | 6.30 |
| 47 | FX | 41.699 | -83.628 | 28.283 | 43.279 | 24.895 | Apis mellifera | 2.67 |
| 48 | FX | 41.699 | -83.628 | 28.283 | 43.279 | 22.077 | Apis mellifera | 2.36 |
| 49 | FX | 41.699 | -83.628 | 28.283 | 43.279 | 27 | Bombus impatiens | 6.09 |
| 50 | FX | 41.699 | -83.628 | 28.283 | 43.279 | 22.132 | Bombus impatiens | 3.30 |
| 51 | FX | 41.699 | -83.628 | 28.283 | 43.279 | 22.589 | Apis mellifera | 4.42 |
| 52 | INT | 41.647 | -83.527 | 59.284 | 61.328 | 21.943 | Apis mellifera | 0.09 |
| 53 | MNR | 41.670 | -83.584 | 40.104 | 45.924 | 19.058 | Apis mellifera | 4.85 |
| 54 | MNR | 41.670 | -83.584 | 40.104 | 45.924 | 36.722 | Bombus impatiens | 3.09 |
| 55 | MNR | 41.670 | -83.584 | 40.104 | 45.924 | 18.725 | Apis mellifera | 0.37 |
| 56 | TUC | 41.637 | -83.654 | 33.143 | 40.848 | 24.915 | Apis mellifera | 0.87 |
| 57 | CC | 41.591 | -83.441 | 16.245 | 32.162 | 27.523 | Bombus impatiens | 4.73 |
| 58 | CC | 41.591 | -83.441 | 16.245 | 32.162 | 34.814 | Bombus impatiens | 6.61 |
| 59 | BWN | 41.699 | -83.589 | 41.200 | 46.614 | 42.465 | Bombus impatiens | 4.92 |
| 60 | CHE | 41.657 | -83.549 | 73.297 | 58.649 | 95.481 | Bombus impatiens | 5.21 |
| 61 | UT | 41.659 | -83.621 | 42.991 | 39.053 | 23.102 | Apis mellifera | 0.80 |
| 62 | BWN | 41.699 | -83.589 | 41.200 | 46.614 | 61.655 | Bombus impatiens | 2.86 |
| 63 | MNR | 41.670 | -83.584 | 40.104 | 45.924 | 31.541 | Bombus impatiens | 16.11 |
| 64 | TUC | 41.637 | -83.654 | 33.143 | 40.848 | 23.84 | Apis mellifera | 0.81 |
| 65 | UT | 41.659 | -83.621 | 42.991 | 39.053 | 23.946 | Apis mellifera | 1.97 |
| 66 | CHE | 41.657 | -83.549 | 73.297 | 58.649 | 36.159 | Bombus impatiens | 5.76 |
| 67 | COL | 41.663 | -83.483 | 20.238 | 44.064 | 46.352 | Bombus impatiens | 6.30 |
| 68 | UT | 41.659 | -83.621 | 42.991 | 39.053 | 21.7 | Apis mellifera | 3.96 |
| 69 | CC | 41.591 | -83.441 | 16.245 | 32.162 | 5.791 | Agapostemon sericeus | 25.25 |
| 70 | CC | 41.591 | -83.441 | 16.245 | 32.162 | 5.962 | Agapostemon sericeus | 23.45 |
| 71 | BU | 41.548 | -83.671 | 16.840 | 29.589 | 16.911 | Bombus impatiens | 5.35 |
| 72 | WW | 41.584 | -83.590 | 19.448 | 27.120 | 5.041 | Agapostemon sericeus | 22.88 |
| 73 | ZP | 41.618 | -83.578 | 53.060 | 43.301 | 13.093 | Agapostemon sericeus | 26.64 |
| 74 | MNR | 41.670 | -83.584 | 40.104 | 45.924 | 6.73 | Agapostemon sericeus | 23.48 |
| 75 | CHE | 41.657 | -83.549 | 73.297 | 58.649 | 24.328 | Apis mellifera | 0.55 |
| 76 | COL | 41.663 | -83.483 | 20.238 | 44.064 | 6.51 | Agapostemon sericeus | 25.65 |
| 77 | COL | 41.663 | -83.483 | 20.238 | 44.064 | 6.073 | Agapostemon sericeus | 20.98 |
| 78 | COL | 41.663 | -83.483 | 20.238 | 44.064 | 5.521 | Agapostemon sericeus | 28.82 |
| 79 | CC | 41.591 | -83.441 | 16.245 | 32.162 | 7.685 | Agapostemon sericeus | 32.59 |
| 80 | COL | 41.663 | -83.483 | 20.238 | 44.064 | 5.247 | Agapostemon sericeus | 28.16 |
| 81 | FTU | 41.611 | -83.619 | 47.690 | 39.347 | 10.618 | Agapostemon sericeus | 27.65 |
| 82 | CC | 41.591 | -83.441 | 16.245 | 32.162 | 3.649 | Agapostemon sericeus | 30.50 |
| 83 | COL | 41.663 | -83.483 | 20.238 | 44.064 | 10.287 | Agapostemon sericeus | 28.02 |
| 84 | CC | 41.591 | -83.441 | 16.245 | 32.162 | 2.789 | Agapostemon sericeus | 37.53 |
| 85 | CC | 41.591 | -83.441 | 16.245 | 32.162 | 6.249 | Agapostemon sericeus | 31.02 |
| 86 | COL | 41.663 | -83.483 | 20.238 | 44.064 | 10.393 | Agapostemon sericeus | 27.17 |
| 87 | TUC | 41.637 | -83.654 | 33.143 | 40.848 | 8.481 | Agapostemon sericeus | 28.25 |
| 88 | BWN | 41.699 | -83.589 | 41.200 | 46.614 | 2.105 | Agapostemon sericeus | 29.13 |
